# Modulation of deoxycytidine kinase (dCK) and glycogen synthase kinase (GSK-3β) by anti-CD20 (rituximab) and 2-chlorodeoxyadenosine (2-CdA) in human lymphoid malignancies

**DOI:** 10.1186/2162-3619-3-31

**Published:** 2014-12-19

**Authors:** Ayad M Al-Katib, Amro Aboukameel, AbdulShukkur Ebrahim, Frances WJ Beck, Samuel E Tekyi-Mensah, Ali Raufi, Yasin Ahmed, Mary Mandziara, Zyad Kafri

**Affiliations:** Lymphoma Research Laboratory, Wayne State University School of Medicine, 540 East Canfield, room #8229, Detroit, MI 48202 USA; Department of Pathology, St John Hospital and Medical Center, Detroit, USA; Van Elslander Cancer Center, Grosse Pointe Woods, MI USA; Quest Diagnostics, 4225 E Fowler Avenue, Tampa, FL 3367-2026 USA

**Keywords:** Lymphoma, Deoxycytidine kinase, Glycogen synthase kinase, 2-chlorodeoxyadenosine (2-CdA), Anti-CD20 (Rituximab)

## Abstract

**Background:**

The combination of rituximab and 2-CdA is an effective therapy for B-cell tumors. However, the molecular mechanisms and enzymatic pathways involved in the interaction between the two agents are not fully understood. In this study, we provide molecular evidence for positive interaction between these two agents with resultant therapeutic benefit.

**Methods:**

Efficacy of the R-2CdA regimen was evaluated in thirteen patients with B-cell tumors (9 CLL; 3 WM and 1 FL), in vitro against 3 lymphoma cell lines and in a xenograft mouse model. Treatment-induced changes involving phenotype, kinase activity and protein expression were assessed in vitro and in the mouse xenograft tumors. The interaction between RTX and 2-CdA was analyzed using the multiple comparison method, Tukey’s honestly significant difference (HSD). For the clinical and animal data, survival functions were estimated using the Kaplan-Meier method and compared by the log-rank test. P-values <0.05 were considered statistically significant. All statistical analyses were evaluated using GraphPad Prism 4 (San Diego, CA).

**Results:**

9 of 12 (75%) evaluable patients responded to the R-2-CdA regimen with median duration of response of 34 months. Median survival of patients from diagnosis and from completion of R-2-CdA treatment was 13.3 and 7.9 years, respectively. In vitro, the combination was effective in all 3 cell lines of lymphomas but with higher sensitivity in the follicular lymphoma cell line. The combination was also effective in the WSU-WM-SCID xenograft model with dose-dependent response and synergistic benefit. All animals were tumor-free for up to 120 days post 2 cycles of this regimen. Rituximab induced activation of deoxycytidine kinase (dCK), p38 mitogen activated protein kinase (p38MAPK) and glycogen synthase kinase-3β (GSK-3β) in the xenograft WSU-WM tumors. Chemical inhibition of p38MAPK led to inhibition of the GSK-3β phosphorylation suggesting that GSK-3β is regulated by p38MAPK in this model.

**Conclusion:**

Collectively, our studies show concordance between the activity of R-2-CdA in vitro, in human and in WSU-WM xenograft model attesting to the validity of this model in predicting clinical response. Modulation of dCK and GSK-3β by rituximab may contribute to the positive therapeutic interaction between rituximab and 2-CdA.

## Background

The anti-CD20 antibody (Rituximab, RTX) represents the most significant advancement in the treatment of B-cell lymphoma and chronic lymphocytic leukemia in the past 30 years [1]. It has activity as single agent in relapsed low-grade follicular lymphoma (FL) which was the basis for its approval by the US Food and Drug Administration (FDA) as therapeutic agent for FL [2]. It has also been shown to prolong progression-free survival of FL patients when used in different maintenance programs following initial chemotherapy [3–6]. The most significant contribution of this agent, however, was its ability to enhance the anti-lymphoma effects of cytotoxic chemotherapy regimens used in the treatment of B-cell lymphoma. Although its initial approval was in FL, it was found to enhance efficacy of chemotherapy in all major types of B-cell lymphoma including FL, diffuse large B-cell lymphoma (DLBCL) and mantle cell lymphoma (MCL) [7]. Since the concept of combining RTX with cytotoxic chemotherapy agents was introduced [8], RTX-chemotherapy combinations have become a standard of care for B-cell lymphoma world-wide [9–11]. Recent data has shown that even transformed low grade lymphoma known to have very poor prognosis, has improved outcome in the rituximab era [12].

Despite its proven activity, the mechanisms of action of RTX are not fully understood. Direct signaling, complement-dependent cytotoxicity (CDC) and antibody-dependent cell-mediated cytotoxicity (ADCC) all appear to play a role in RTX anti-lymphoma effect [13]. Each of these mechanisms appears to be differentially more important in different settings depending on cell type, immune system status and other factors [14]. Direct binding of RTX to CD20 triggers a number of signaling pathways including apoptosis, activation (or downregulation) of protein kinases, phosphatases and caspases [15]. However, the molecular basis for chemotherapy-enhancing effect of RTX remains largely unknown.

In this study, we analyzed the activity of the R-2-CdA regimen where RTX (R) is given in combination with 2-chlorodeoxyadenosine (2-CdA) in patients with low grade B-cell tumors, in a mouse xenograft model for human Waldenstrom’s macroglobulinemia and in vitro studies. The nucleoside analogue 2-CdA as single agent has activity in low grade non-Hodgkin’s lymphoma (NHL) and in chronic lymphocytic leukemia/small lymphocytic lymphoma (CLL/SLL) when used as first line therapy [16, 17] or as ‘salvage’ therapy following relapse of disease [18]. This agent has also been used in combination with RTX alone or with RTX plus other agents such as cyclophosphamide [19–21] with superior results to that of 2-CdA alone [22–24].

Results of studies reported here reveal that R-2-CdA regimen has significant activity in relapsed low grade NHL. Moreover, we present evidence to show that RTX can modulate specific protein kinases such as deoxycytidine kinase (dCK) and glycogen synthase kinase 3β (GSK3β) which are new findings. We also demonstrated that p38 mitogen-activated protein kinase (p38 MAPK) can regulate GSK3β. These findings provide some insight into the mechanisms of RTX-induced enhancement of chemotherapy effects.

## Material and methods

### Clinical study

We retrospectively reviewed records on 13 patients who were treated with 2-CdA and RTX combination (R-2-CdA) at the Van Elslander Cancer Center during the period of January 2005-January 2013. The study was approved by St. John Hospital & Medical Center’s institutional review board. Patient selection was based on medical records showing use of R-2-CdA combination therapy in patients with B-Cell lymphoma. Primary end points of the study were response rate, response duration and survival. Secondary endpoints included toxicity of the regimen. Rituximab was administered intravenously (iv) on day one using standard dose of 375 mg/m^2^ and a standard protocol. Patients were premedicated with acetaminophen (1000 mg orally X1), diphenhydramine (Benadryl®) iv at a dose of 25 mg repeated if necessary, and methylprednisolone sodium succinate, USP (Solu-medrol®) iv at a dose of 125 mg repeated if necessary for rituximab-related infusion reaction. Rituximab infusion was initiated at a low rate of 50 mg/m^2^ and doubled every thirty minutes, to a maximum rate of 400 mg/m^2^ provided there were no adverse reactions and patient’s vital signs were stable. At the completion of rituximab infusion on day 1, a 2-CdA pump was hooked up to a central venous access (MediPort) and 2-CdA was delivered at a dose of 0.1 mg/kg/day as continuous infusion for 7 consecutive days. The treatment was repeated in 28-day cycles but delayed when necessary to allow for recovery of cell counts or other toxicities. In one patient, however, 2-CdA was delivered subcutaneously due to lack of venous access. In another patient, 2-CdA was delivered daily over 5 days as iv infusion over 2 hours according to Nagai et al. [25]. Toxicity was graded according to the US National Cancer Institute using the Common Terminology Criteria for Adverse Events (CTCAE) version 4.03. In general, toxicity is graded as mild (grade 1), moderate (grade 2), severe (grade 3) or life threatening (grade 4). Death is considered grade 5.

### In vitro studies

#### Cell lines

The WSU-WM, WSU-DLCL2 and the WSU-FSCCL cell lines, used in this study, were established at Wayne State University (WSU) and their characteristics have been previously published [26–29]. The WSU-WM was established from a patient with Waldenstrom’s macroglobulinemia; WSU-DLCL2 represents diffuse large B-cell lymphoma (DLBCL), and WSU-FSCCL was established from a patient with follicular lymphoma, grade 1 according to the current World Health Organization (WHO) classification. All three cell lines are EBV negative and are maintained in a liquid culture medium consisting of RPMI 1640 supplemented with 10% fetal bovine serum (FBS), 1% L-glutamine and 1% penicillin-streptomycin. The phenotypic and cytogenetic characteristics of the cell lines are checked periodically to determine stability.

### Cell growth and viability

WSU-WM, WSU-FSCCL and WSU-DLCL2 cells were seeded at 2.0 × 10^5^ cells/ml in a 24 well plate (in triplicate). 2-CdA (50 nM), RTX (100 μg) individually or in combination were added to respective wells. Cultures were incubated for 96 h at 37°C and 5% CO_2._ Viability and growth inhibition were determined each day using 0.4% trypan blue (Sigma) exclusion.

#### Inhibition of p38MAPK using SB203580

WSU-WM cells were seeded at 1 × 10^6^ cells/5 ml in T25 flask (in triplicate). 2-CdA (50 nM), RTX (100 μg) or vehicle (DMSO and RPMI 1640 medium) added to respective flasks. Cultures were incubated for 96 h at 37°C and 5% CO_2_. After 48 h 2-CdA or RTX treatment, p38MAPK specific inhibitor, SB203580 (Selleck Chemicals, USA) was added at 30 μM. Cells were counted at 72 h and 96 h in the presence or absence of inhibitor. At selected time points, cells were collected and protein expression was evaluated by Western blot analysis as described below.

#### Western blotting

Control and drug-treated WSU-WM cells were sonicated and collected by centrifugation, washed twice with sterile PBS, and solubilized in RIPA lysis buffer (ThermoScientific, Rockford, IL) consisting of a cocktail of protease and phosphatase inhibitors. Total cell protein was quantified by BCA method (Pierce; Thermo Scientific). 50 μg aliquots of protein were fractionated onto 4-20% Tris-Glycine SDS-PAGE gels, then transferred onto PVDF membrane. Membranes were blocked in PBS with 5% (w/v) fat-free milk powder and probed with primary antibodies to p-p38MAPK (Thy 182) and p-GSK3β (Ser 9) (Cell signalling Technology, Danvers, MA). GAPDH (Sigma) was used as the internal control. The secondary antibodies were anti-mouse or anti-rabbit conjugated to HRP (Jackson ImmunoResearch). After further extensive washing in PBS-T, the blots were developed using enhanced chemiluminescence substrate reagents (Thermo Scientific).

#### Pre-clinical in vivo studies

##### WSU-WM-SCID mouse xenograft model

This subcutaneous model was initiated by injecting WSU-WM cells (10^7^) subcutaneously (sc) into the flank areas of 3–4 week old mice with severe combined immune deficiency (SCID) as previously described [26, 27]. Palpable tumors developed in about 2 weeks. Tumors were propagated serially in-vivo by transplanting tumor fragments into the flanks of a new batch of animals. CB17 and ICR SCID mice (Taconic Farms, Germantown, NY) have both been used with equal success for in-vivo WSU-WM tumor growth. No preconditioning of animals with radiation or cyclophosphamide was necessary prior to transplantation. Animals were kept in a protected environment and were euthanized when tumor weight reached ~2000 mg (~10% of body weight) to avoid animal discomfort. All animal experiments were done according to Institutional Animal Care and Use Committee (IACUC)-approved protocol of WSU.

#### Pre-clinical efficacy trial studies

WSU-WM tumor fragments were transplanted sc bilaterally into the flanks of ICR-SCID mice as described above. Once palpable tumors were established, animals were removed randomly and assigned to different interventions: Control (PBS); RTX; 2-CdA; and RTX plus 2-CdA. Two different experiments were conducted sequentially with different endpoints and doses as described below:

##### Experiment 1

In this experiment, 40 ICR-SCID mice bearing subcutaneous WSU-WM tumors in both flanks, developed as above, were used. Groups of 10 animals were removed randomly and assigned to different interventions: Control; RTX; 2-CdA; and RTX plus 2-CdA. RTX was administered iv via tail vein daily (QD) X 5 consecutive days (1–5) at 40 mg/kg/day. 2-CdA was administered concurrently at 20 mg/kg/day, sc QD, days 1–5. The combination group received both agents at the same dose and route, ie: RTX at 40 mg/kg/day iv and 2CdA at 20 mg/kg/day sc both given days 1–5. Treatment began 7 days after tumor implantation. Three animals out of each group were randomly selected and tumors removed for analysis by Western blotting and flow cytometry as described below. For the remaining 7 animals in each group, survival was the endpoint of study. To determine survival, animals were monitored for tumor growth and toxicity. Tumor-bearing animals were euthanized when total palpable tumor weight approached or exceeded 2000 mg (~10% of the body weight) to avoid discomfort. Survival is calculated as the interval (in days) between first day of treatment and day of euthanasia. The experiment was terminated at day 80 where all remaining animals were euthanized whether or not they were carrying tumors.

##### Experiment 2

The design of the second experiment was to evaluate the efficacy of increased dose of RTX, 2-CdA and the combination in the WSU-WM model. In this experiment, 32 ICR-SCID mice were utilized. WSU-WM tumor fragments were transplanted sc as above. Animals were randomly assigned to one of 4 groups, 8 animals each: Control, RTX, 2-CdA and RTX plus 2-CdA. Treatment began 7 days after tumor implantation. RTX was administered iv through tail vein daily for 5 days at 150 mg/kg/day. 2-CdA was administered at 30 mg/kg/day, sc QD x 5. The 2-CdA dose used here is the maximum tolerated dose (MTD) in SCID mice as previously determined in our laboratory [30]. The RTX dose and schedule were based on previously published work from our laboratory [21]. The combination treatment group received the same doses of RTX and 2-CdA daily for 5 days followed by one week rest and a second cycle of same doses. End points of this experiment were tumor response and 120-day tumor-free survival. The National Cancer Institute (NCI) criteria for anti-tumor activity were used for drug efficacy assessment. Tumor weights were estimated from two-dimensional measurements: Tumor weight (mg) = (A × B^2^)/2, where “A” and “B” are the tumor length and width in mm respectively. Tumor growth inhibition (T/C) is the median tumor weight of the treated group (T) divided by the median tumor weight of the control group (C) at a time when the median tumor weight in the control group reached approximately 700 mg. Tumor growth delay (T-C) is the difference between the median time, in days, required for the treatment group tumor (T) to reach 900 mg and the median time in days for the control group tumor (C) to reach the same weight. The Log10 tumor cell kill total (gross) = (T-C)/ (3.32) (Td) where Td is the time in days required for the tumor to double its weight during the exponential growth phase. Cure is defined as mice being tumor-free for 120 days after therapy.

#### Phenotypic analysis of WSU-WM-SCID xenografts by flow cytometry

Six female ICR-SCID mice (Taconic Farms, Germantown, NY), 3–4 weeks old were transplanted subcutaneously bilaterally with tumor fragments (20–30 mg pieces) of WSU-WM tumor as described above. One week later, when palpable tumors developed (~140 mg), mice were randomly separated into two groups of equal tumor burden. Group 1 was treated with RTX at 150 mg/kg/injection (via tail vein, daily for 5 days). Group 2 (control) received diluent for 5 days. Three days post last injection, animals in treated group that had developed re-growth of tumors after initial shrinkage were euthanized, their tumors removed and dissected into single cell suspension, then subjected to phenotypic analysis by flow cytometry. For each RTX-treated animal studied, similarly processed tumors from diluent-treated animals were analyzed as control. One hundred μl of each tumor cell suspension (approximately 1 × 10^6^ cells/ml) were placed into 12 × 75 plastic tubes, washed three times with 1% BSA-PBS. Supernatant was decanted, FITC- conjugated primary mouse anti-human antibodies were added to each tube. Cells were then washed after 30 minutes of incubation in the dark at 4°C before analysis using FACScan. Results were compared with tumors obtained from the control group. The following antibodies were purchased from DAKO Corporation (Carpenteria, CA) anti -CD19, −CD20, −CD10, −CD22, −IgM, −lambda, −CD55, −CD59. For anti-Rituximab staining, the same procedures were applied using anti-C2B8 antibody (clone directed against Rituximab antibody) generously provided by Genentech Corp (San Francisco, CA).

#### dCK assay

For these experiments, 18 WSU-WM tumor-bearing SCID mice were separated into 3 groups. Within each group of 6 animals, 3 received 1, 3, or 5 daily injections of RTX and the other 3 received diluent (as control). At the end of the treatment, animals were euthanized, tumors dissected and mechanically dissociated into single cell suspension using steel mesh. After washing, cells were processed for dCK enzyme activity assay as previously described [30]. Briefly, harvested cells were washed with PBS × 2, aliquoted at a concentration of 10^7^ cells/ml/sample in sonication buffer (50 mM Tris, 2 mM DTT, 0.5 mM PMSF, 10% Glycerol and 40 ml deionized H_2_0) and stored at -80°C until assayed. DCK was determined using [8-^3^H]-2-CdA as a substrate and dCK activity was calculated as pmol of phosphorylated 2-CdA generated per mg protein per minute.

### Statistical analysis

For all cell line experiments, data were compiled from at least three independent triplicate experiments performed on separate cultures. The interaction between RTX and 2-CdA was analyzed using the multiple comparison method called Tukey’s honestly significant difference (HSD). Statistical significance (P < 0.05) id denoted on graphs as *. For the clinical data and animal experiments, survival functions were estimated using the Kaplan-Meier method and compared by the log-rank test. P-values <0.05 were considered significant. All statistical analyses were evaluated using GraphPad Prism 4 (San Diego, CA).

## Results

### Efficacy of R-2-CdA in Patients with indolent B-cell tumors

**Figure 1:**
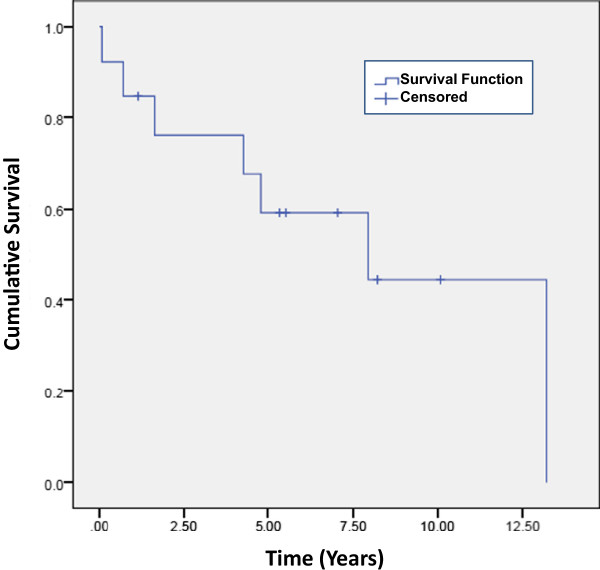
**Survival of patients treated with the R-2-CdA regimen (n = 13).** Kaplan-Meier overall survival from end of therapy with R-2-CdA to last follow up (censored) or death. Median survival is 7.9 years.

Thirteen patients were included in this study between January 2005 and January 2013. As shown in Table 1, there were 7 males and 6 females; median age at diagnosis was 48 (Range: 39–71), while the median age at time of R-2-CdA treatment was 59 (Range 40–76). Nine patients had a diagnosis of SLL/CLL; 3 were WM and one with follicular lymphoma grade 1/2. All patients except the follicular lymphoma patient were previously treated with standard therapy consisting of rituximab and cytotoxic chemotherapy. (Median = 3 regimens, range 2–7). Patients received a median of 2 cycles of R-2-CdA therapy (range, 1–5 cycles). Seventy seven percent (10/13 patients) responded; seven (54%) with complete response (CR) and 3 (23%) with partial response (PR); the remaining 3 patients had progressive disease (PD). The median duration of response to R-2-CdA therapy was 34 months (range 8-118+). Overall survival from diagnosis to death or last follow up was 13.3 years and survival from cessation of R-2-CdA treatment was 7.9 years (Figure 1). Major toxicity was hematologic (Table 1), however, weight loss and fatigue were also common. There were no treatment-related deaths.

**Table 1:** **Clinical data**

Patient demographics					
Total number of patients	13				
Male: Female	7:6				
Median Age at Diagnosis in years (range)	48 (39–71)				
Median Age at R-2CdA treatment (range)	59 (40–76)				
**Histology**					
Chronic lymphocytic leukemia/small	9				
Lymphocytic lymphoma (CLL/SLL)					
Waldenstrom’s Macroglobulinemia (WM)	3				
Follicular lymphoma Grade I/II	1				
**Stage**					
Rai Stage IV	6/8				
Ann Arbor (AA) Stage IV	4/4				
Not determined	1				
**Prior therapy**					
Previously treated	12				
Median number of prior regimens (range)	3 (2–7)				
**Response**					
Total response rate (RR) (number/percent)	10/13 (77%)				
Complete response (CR)	7/13 (54%)				
Partial response (PR)	3/13 (23%)				
Progressive disease (PD)	3/13 (23%)				
Median duration of response (range)	34 months (8-118+)				
**Common toxicity**	**Grade**				
	**Total %**	**1**	**2**	**3**	**4**
Anemia	8 (62%)	6	1	1	
Thrombocytopenia	8 (62%)	5	1	2	
Neutropenia	7 (54%)	2	3	2	
Fatigue	7 (54%)	2	3	2	
Weight loss	5 (38%)	3	2

### In vitro cytotoxicity

**Figure 2:**
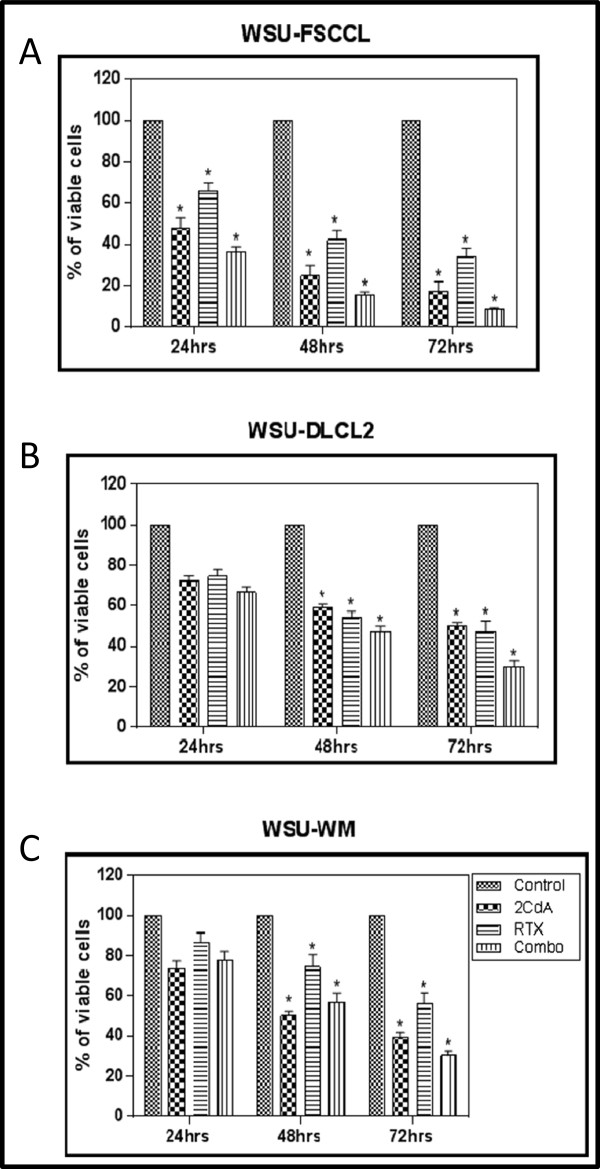
**Effect of rituximab (RTX) and 2-CdA alone or in combination (combo) on lymphoma cell lines in vitro.** Cell viability was measured by 0.4% trypan blue exclusion assay over a 72 h incubation period [mean and standard error means (SEM)]. Cell lines used were: **(A)** WSU-FSCCL (follicular lymphoma); **(B)** WSU-DLCL2 (diffuse large B-cell lymphoma); and **(C)** WSU-WM (Waldenström’s macroglobulinemia). Cells were exposed to each agent at the beginning of culture (time 0) with 2-CdA at 50 nM, RTX 100 μg, or both agents at the same concentration. *indicates significant difference compared with control (p < 0.05).

The three lymphoma cell lines used represent the spectrum of B-cell lineage tumors: (a) WSU-WM (plasmacytoid type); (b) WSU-FSCCL (follicular low grade lymphoma); (c) WSU-DLCL2 (diffuse large B-cell lymphoma). In all three cell lines, exposure to both agents individually and in combination caused progressive decrease in viable cells in culture over a period of 72 h (Figure 2). The WSU-FSCCL cells were the most sensitive where s significant decrease in cell viability was evident in the first 24 h compared with control. In general, the combination of the two agents exhibited greater cell toxicity compared with each individual agent alone. However, using multiple comparison method (HSD), the difference between the combination and single agents were not significant in the WSU-WM and WSU-DLCL2 cells (p > 0.05). In the WSU-FSCCL cells, the difference between the combination and RTX was significant (p = 0.0003 at 24 h; 0.0002 at 48 h; and 0.004 at 72 h) whereas the combination versus 2-CdA was not significant (p = 0.99-1.0).

### Efficacy of R-2-CdA in the WSU-WM-SCID mouse xenograft model

#### Experiment 1

**Figure 3:**
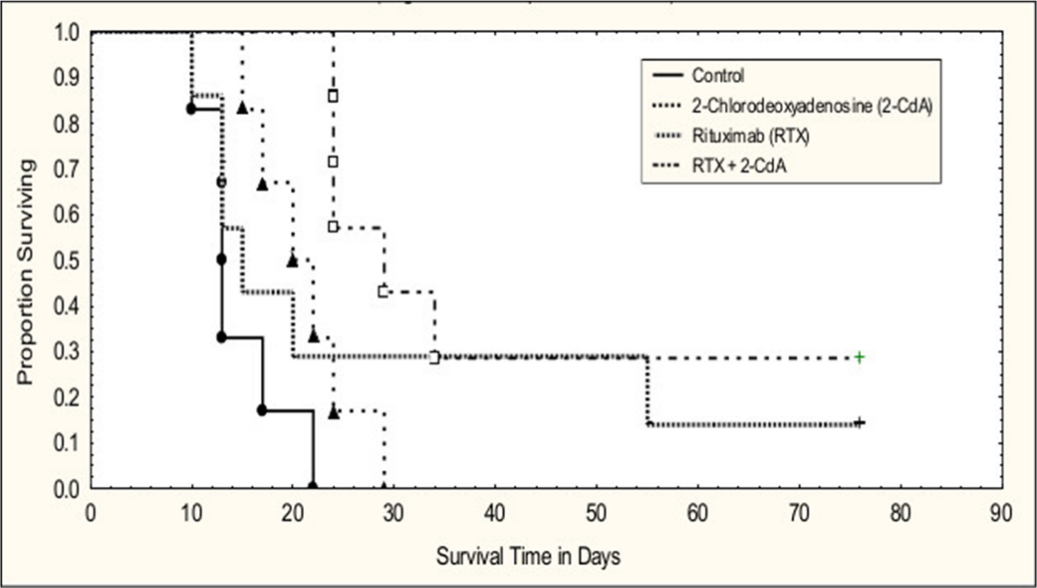
**Survival of WSU-WM-bearing ICR-SCID mice treated with 2-CdA, RTX alone or in combination.** Log-Rank Test showing the combination (RTX +2-CdA) to be statistically superior to single agents (p = 0.01007).

As shown in Figure 3, all animals in the control group were euthanized within the first 22 days of treatment. 2-CdA treatment showed a modest but non-significant improvement in survival compared with control. This agent was effective for a short period of time in delaying tumor progression compared with control. However, tumors grew rapidly once the disease progressed and all remaining animals were euthanized within 30 days of treatment. In contrast, in animals treated with RTX, tumor development was delayed in one animal of the RTX-treated group (day 45) and another animal survived to the end of the experiment and was tumor-free. The combination of the two agents was clearly more effective (p = 0.01) compared with single agents and 2 of the 7 animals within this group were tumor-free at the end of the experiment.

#### Experiment 2

In this experiment, we tested a higher dose-intensity of the R-2-CdA regimen. As summarized in Table 2, both RTX and 2-CdA exhibited modest activity as single agents even at higher doses. At a median tumor weight in the control group of 700 mg, there was 30% and 36.9% tumor growth inhibition caused by RTX and 2-CdA treatment, respectively (<42% is considered active). Tumor growth was delayed by 13 and 14 days in the RTX and 2-CdA treated groups, respectively compared with control. Activity was also modest by the log_10_Kill measure (1.9 and 1.33 for RTX and 2-CdA, respectively). None of the animals in the single agent-treated groups survived to the end of the experiment, ie, all developed tumors and were euthanized. However, in the combination group, all animals were tumor-free at the end of the experiment (120 days) as confirmed by pathologic examination at necropsy. There were also no treatment-related deaths in this study using increased doses of both RTX and 2-CdA.

**Table 2:** **Efficacy of rituximab and 2-CdA in the WSU-WM-SCID xenograft model**

Agent	Dose/(mg/kg), schedule	Route	Mice	T/C (%)	T-C Days	Log _10_ kill	Cure
Control	Diluent, QD × 5	i.v.	8	100	0	0	0/8
Rituximab	150 mg, QD × 5	i.v.	8	30	13	1.9	0/8
2-CdA	30 mg, QD × 5	s.c.	8	36.9	14	1.33	0/8
(RTX + 2-CdA)	(150 + 30),QD × 5	(i.v. + s.c.)					
Diluent	Diluent, QD × 5	i.v.	8	16.9	N/A	N/A	8/8
(RTX + 2-CdA)	(150 + 30),QD × 5	(i.v. + s.c.)					

### Phenotypic analysis of residual WSU-WM xenograft tumors following RTX therapy

**Figure 4:**
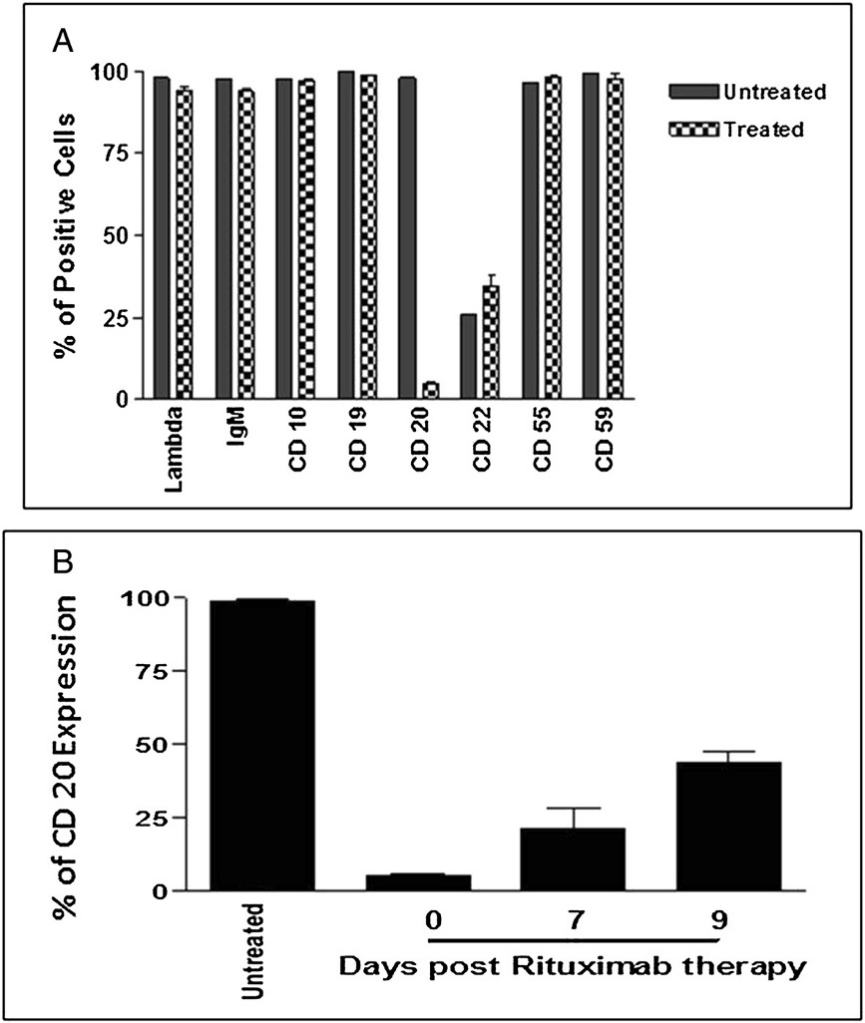
**Flow cytometric analysis of WSU-WM xenograft tumors. A**. Phenotype of tumor cells in control (untreated) and following rituximab therapy, **B**. CD20 expression in residual tumors removed at days 7 and 9 post completion of RTX therapy compared with day of completion of therapy (day 0) and with tumors of untreated animals (untreated).

**Figure 5:**
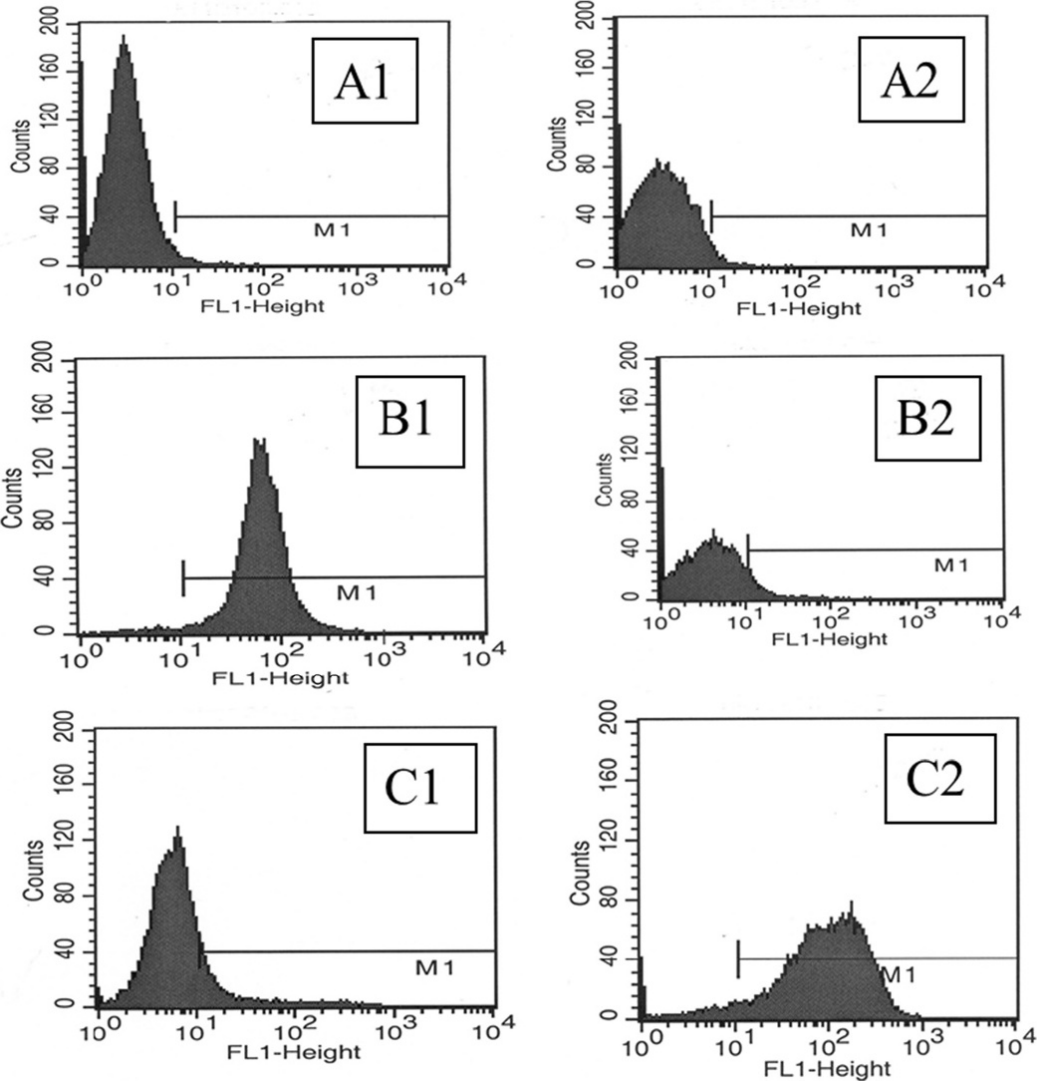
**Detailed analysis of CD20 expression in residual tumor cells following treatment of animals with rituximab.** A1 – Isotype control for CD 20; A2 – Isotype control for anti-rituximab (anti-C2B8); B1– CD 20, untreated animal (positive expression); B2 – Anti-C2B8, untreated animal (negative expression); C1– CD 20, RTX-treated animal (negative expression); C2 – Anti-C2B8 in RTX-treated animal (positive expression).

In order to determine the effect of RTX therapy on the WM phenotype, residual tumors (after RTX therapy) were examined by flow cytometry. As shown in Figure 4A, there was no significant change in WM phenotype in RTX-treated animals compared with control, except for CD20 and CD22 antigens. The RTX target antigen CD20 became undetectable in 97-98% of cells, whereas CD22 showed some increase in the RTX-treated tumors compared with control (Figure 4A). CD20 antigen expression became detectable again within a few days after completion of RTX therapy. By day 9 post-therapy, ~40% of tumor cells were CD20 + (Figure 4B). To determine whether CD20 negativity represented a subpopulation of RTX-resistant cells or antigen masking, i.e. false negative, additional experiments were conducted. Using anti-RTX antibody (anti-C2B8 clone), we demonstrated that WSU-WM xenograft tumor cells of RTX-treated animals were positive for C2B8, indicating that RTX was still bound to the CD20 antigen (Figure 5). These results indicate that the reported “negative” expression of CD20 in RTX-treated xenografts is due to the antigen being masked by RTX rather than the lack of CD20 antigen expression.

### RTX therapy increases dCK activity in WSU-WM tumors

**Figure 6:**
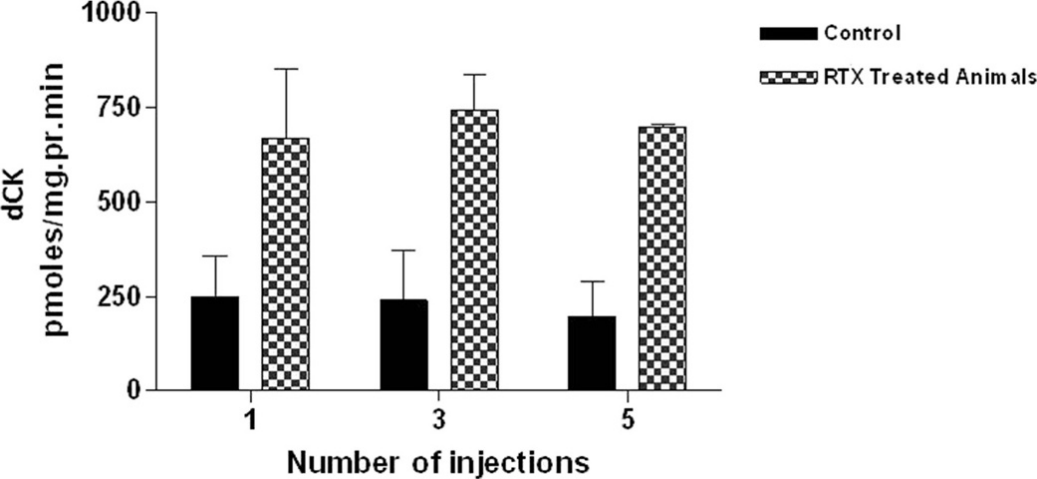
**Deoxycytidine kinase activity (dCK) in WSU-WM tumors removed from untreated SCID mice (control) or following 1, 3, or 5 injections of RTX as described in the Method section.** Each bar represents mean and SEM of 3 animals.

dCK is a key enzyme for the intracellular phosphorylation of nucleoside analogues, in this case, 2-CdATP. We therefore sought to determine if combining RTX with 2-CdA has any impact on dCK activity. As shown in Figure 6, dCK activity in WSU-WM tumors obtained from control mice ranged from 34.7 to 389.26 pmoles/mg/protein/min (Mean ± S.D. = 226 ± 153) and did not increase with time. In contrast, dCK activity in tumors obtained from RTX-treated mice increased relative to the control and became statistically significant as the number of RTX injections increased (RTX one injection = 669 ± 319, p = 0.128; RTX three injections = 743 ± 165, p = 0.013; RTX five injections = 698 ± 13, p = 0.00004). This suggests that multiple injections or a sequential dosing schedule may be necessary to achieve the greatest effect on dCK activity.

### Modulation of p38MAPK and GSK3β by RTX and 2-CdA

**Figure 7:**
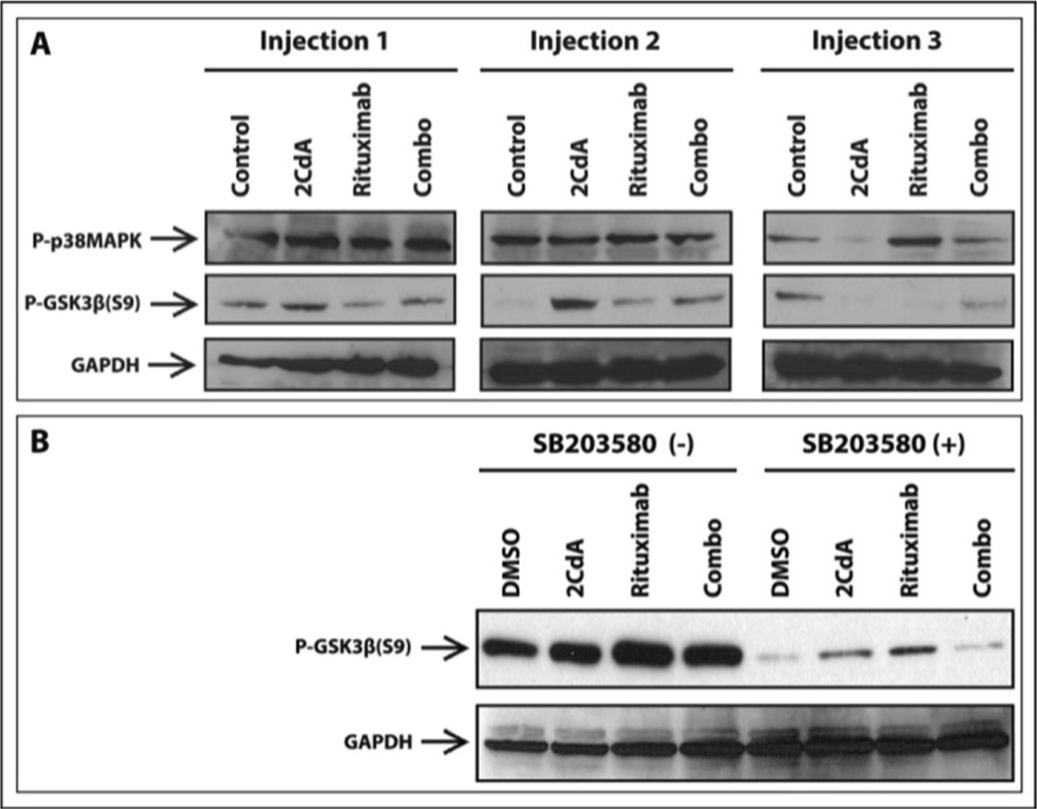
**p38MAPK and GSK3** **β** **protein expression by Western blots in the WSU-WM model. A**. Expression in xenograft tumors taken from SCID mice following treatment with 1, 2 or 3 injections with rituximab, 2-CdA or the two agents (combo). 50 μg total tumor tissue lysates were subjected to Western blot for detection of phospho-p38MAPK, phospho-GSK3β using GAPDH as a loading control; **B**. Effect of chemical inhibition of p38MAPK on GSK3β expression in vitro. WSU-WM cells in culture were exposed to 2-CdA (50nM), RTX (100 μg), or combo (2-CdA + RTX). 48 h later, DMSO (vehicle) or p-38MAPK inhibitor SB203580 (30 μM) were added and cultures were maintained for an additional 48 h. At the end of incubation, lysates were collected and subjected to immunoblotting using GAPDH as loading control.

Changes in the p38MAPK and GSK3β were evident after the 3rd injection of mice carrying the WSU-WM tumors with RTX and 2-CdA. There was a sustained activation of p38MAPK (p-p38MAPK) in tumors of the RTX-treated animals and a decrease in phosphorylated GSK3β (Figure 7A). This finding suggested a relationship between p38MAPK and GSK3β which was investigated further in vitro. Under different experimental conditions, we induced activation of p38MAPK by RTX and 2-CdA (for 48 h) then blocked it chemically by SB203580. Such experiment revealed that chemical inhibition of RTX- and 2-CdA-induced activation of p38MAPK significantly reduced phosphorylation of GSK3β (Figure 7B). This experiment indicated that GSK3β can be regulated by p38MAPK in this tumor model. Together with the animal data, these results suggest that p38MAPK can be a GSK3β kinase.

## Discussion

Although low grade B-cell tumors in humans remain incurable, availability of alternative treatment regimens have contributed to prolongation of survival of these patients. This observation is illustrated in our study where there was 11 years lapse time in median age between diagnosis (median age 48 years) and R-2-CdA treatment (59 years, Table 1). Moreover, the median survival of our patients from completion of R-2CdA treatment was 7.9 years (Figure 1). The R-2-CdA regimen exhibited impressive activity as salvage regimen in our advanced-stage, heavily pretreated patient population where 77% of patients responded with a median duration of response of 34 months.

Each individual agent in the R-2-CdA regimen individually has activity in low grade B-cell tumors. When used as salvage therapy in previously treated patients with low grade (indolent) NHL, 2-CdA produced 35% complete response (CR) and 54% partial response (PR) rate [16]. In another study, the overall response rate (ORR) was 43% [18]. As frontline therapy for CLL/SLL, 2-CdA produced 25% CR and 60% PR [17]. Rituximab as single agent, on the other hand produced 67% and 46% ORR when given to previously untreated and as salvage therapy for patients with follicular lymphoma (FL), respectively [6]. In CLL/SLL, rituximab monotherapy upfront produced 58% ORR [31].

Rituximab and 2-CdA have been administered in combination as a two-agent regimen or in combination with additional agents in low grade B-cell tumors. In one study, the R-2-CdA regimen was given to patients with Waldenstrom’s macrgolbulinemia (WM) where the ORR was 89.6% without differences being observed between previously treated and untreated patients [23]. The ORR in this study however included minor responses besides CR and PR. In another study, R-2-CdA produced 15.4% CR and 53.8% PR (ORR =69.2%) in relapsed/refractory patients with low grade NHL [24]. 2-CdA in combination with oral cyclophosphamide therapy resulted in an overall response rate of 84% in previously untreated patients with WM whereas 2-CdA/cyclophosphamide/RTX combination resulted in an overall response of 94% [22]. It is possible that a 2-CdA/RTX combination may yield comparable response rates without the added toxicity of cyclophosphamide [32]. This is supported by the results of another study where there was no difference in efficacy between cyclophosphamide plus 2-CdA compared with 2-CdA alone in CLL/SLL [33]. Whether or not the activity of R-2-CdA regimen is limited to the indolent NHL still remains an open question. Our limited in vitro study suggests that R-2-CdA may have activity in more aggressive tumors like DLBCL albeit lower than that seen in FL (Figure 2).

None of the clinical studies mentioned above addressed possible mechanisms of interaction between RTX and 2-CdA, perhaps due to the limited availability of patient samples for such investigation. In our study, we utilized the WSU-WM-SCID mouse xenograft model as a source of tumor tissue to investigate possible mechanisms of interaction between the 2 agents. We first confirmed the activity of the R-2-CdA regimen in this model (Figure 3) and demonstrated superior value of the combination versus single agent (p = 0.01, Figure 3). We also demonstrated that increased dose-intensity of the R-2-CdA regimen is feasible and can produce higher and more durable responses. Two cycles of the regimen eradicated the xenograft tumors in all animals and were well tolerated (Table 2). This observation suggests that higher number of treatment cycles in human, whenever possible, may produce more durable remissions.

A key question in this study relates to the potential mechanisms of interaction between RTX and 2-CdA. We first analyzed the phenotype of residual WSU-WM xenograft tumors following treatment with RTX. The two molecules identified with RTX resistance (CD55 and CD59 [34]) were brightly positive at baseline in the WSU-WM tumors and were not changed significantly after RTX treatment. However, the most remarkable change in phenotype following RTX treatment was the apparent negative expression of CD20 (Figure 4A). CD20 expression became detectable slowly after RTX treatment was terminated, as shown in Figure 4B. Within 9 days of therapy, CD20 expression was again detectable by flow cytometry in 40% of cells. Using RTX antibody, we proved that the apparent negative expression is due to CD 20 antigen masking by RTX (Figure 5). The return of CD20 expression within several days supports the use of repeated doses of RTX infusion in conjunction with chemotherapy.

The phenotypic studies nullified our previous hypothesis that 2-CdA can eradicate a subpopulation of CD20 (−) B-cell lymphoma that are resistant to RTX hence providing a mechanism of interaction between the 2 agents.

We next argued that enhancing dCK activity can be a mechanism of interaction between RTX and 2-CdA. The dCK enzyme is paramount in the deoxynucleoside salvage pathway and in the activation of numerous nucleoside analogues used in cancer and in antiviral chemotherapy including 2-CdA. Reduction in dCK activity was a major cause of 2-CdA resistance [35]. As shown in Figure 6, RTX treatment induced a 3 fold increase in dCK activity within WSU-WM xenograft tumors with increasing statistical significance as the number injections increased. Although several factors are involved in determining activity of 2-CdA against cancer cells, intracellular phosphorylation of 2-CdA is a necessary step. The drug is phosphorylated by dCK and accumulates in the cells as 2-chlorodeoxyadenosine triphosphate (2-CdA TP) which is the active form [36]. The increase in dCK activity induced by RTX in the xenograft tumors can be one mechanism by which RTX enhances the activity of 2-CdA.

The mechanism by which RTX induces activation of dCK remains elusive. However, we looked at impact of RTX on other kinases relevant to cancer. RTX treatment of WSU-WM-bearing SCID mice induced sustained activation (phosphorylation) of p38MAPK (p-p38MAPK) and dephosphorylation (activation) of GSK3β (Figure 7A) suggesting a possible relationship between these 2 kinases in this model. However, under different experimental conditions in vitro, exposure of WSU-WM cells to RTX and 2-CdA induced phosphorylation of GSK3β. Chemical inhibition of p38MAPK by SB203580 inhibited treatment-induced phosphorylation of GSK3β (Figure 7). To our knowledge, this is the first evidence that GSK3β can be regulated by p38MAPK.

GSK-3, a serine/threonine kinase plays an important role in a variety of cellular processes such as cell proliferation, microtubule dynamics, cell cycle and apoptosis [37]. GSK3β, the key component that regulates canonical WNT/β-catenin and PI3K/Akt signaling pathways, is commonly considered a tumor suppressor in multiple cancers [38]. The activity of GSK3β can be reduced by phosphorylation at Ser-9. Several kinases are able to mediate this modification like p70S6, p90RSK, Akt and PKC [39, 40]. Dysregulation of GSK3β has been implicated in many pathological conditions in man. Within cancer, it was shown to have varying, and sometimes opposing effects. For example, GSK3β was shown to inhibit growth of prostate cancer cells thus acting as a tumor suppressor [41]. In contrast, GSK3β was shown to participate in cell survival in pancreatic cancer hence behaving as a tumor promoter [42]. Moreover, the kinase has dual function where it can activate or inhibit apoptosis [43]. The divergent functions of GSK3β appear to depend on cell type, intracellular pathways triggered by therapeutic agent(s) tested and the experimental conditions. In fact this context-dependent biological effect concept of therapeutic agents in cancer can be generalized. One example is our observation of persistent p38MAPK activation in RTX-treated WSU-WM-SCID xenograft tumors (Figure 7A) which is in contradistinction to previous reports where RTX caused inhibition of p38MAPK in vitro in a diffuse large B-cell lymphoma cell line [44, 45]. Whether GSK3β, p38MAPK, or other protein kinase(s), are involved in dCK activation will require further investigation.

## Conclusions

In conclusion, R-2-CdA regimen can activate multiple protein kinases known to contribute positively to anti-cancer activity. There appears to be interaction between these kinase pathways as demonstrated in the relationship between p38MAPK and GSK3β. Such interaction can be the basis for synergism between RTX and 2-CdA. Determining whether RTX-induced activation of dCK is mediated by its phosphorylation and which kinase pathway involved in such activation needs further investigation.

## Abbreviations

2-CdA: 2-chlorodeoxyadenosine
ADCC: Antibody-dependent cell-mediated cytotoxicity
CLL: Chronic lymphocytic leukemia
CTCAE: Common terminology criteria for adverse events
CDC: Complement-dependent cytotoxicity
dCK: Deoxycytidine kinase
DLBCL: Diffuse large B-cell lymphoma
FBS: Fetal bovine serum
FL: Follicular lymphoma
GSK3β: Glycogen synthase kinase
HSD: Honestly significant difference
IACUC: Institutional Animal Care and Use Committee
MCL: Mantle cell lymphoma
MTD: Maximum tolerated dose
NCI: National Cancer Institute
NHL: Non-Hodgkin’s lymphoma
RTX: Rituximab
R-2-CdA: Rituximab 2-chlorodeoxyadenosine
SCID: Severe combined immune deficiency
SLL: Small lymphocytic lymphoma
sc: Subcutaneously
WM: Waldenström’s macroglobulinemia
WSU: Wayne State University
WHO: World Health Organization.
